# Prevalence of Child-Directed Marketing on Breakfast Cereal Packages before and after Chile’s Food Marketing Law: A Pre- and Post-Quantitative Content Analysis

**DOI:** 10.3390/ijerph16224501

**Published:** 2019-11-15

**Authors:** Fernanda Mediano Stoltze, Marcela Reyes, Taillie Lindsey Smith, Teresa Correa, Camila Corvalán, Francesca R. Dillman Carpentier

**Affiliations:** 1 Hussman School of Journalism and Media, University of North Carolina, Chapel Hill, NC 27599, USA, fmediano@email.unc.edu; 2 Carolina Population Center, University of North Carolina, Chapel Hill, NC 27516, USA; taillie@unc.edu; 3 Institute of Nutrition and Food Technology, University of Chile, 7830489 Santiago, Chile; mreyes@inta.uchile.cl (M.R.); ccorval@gmail.com (C.C.); 4 Department of Nutrition, Gillings School of Global Public Health, University of North Carolina, Chapel Hill, NC 27599, USA; 5 School of Communication, Diego Portales University, 8370109 Santiago, Chile; teresa.correa@udp.cl

**Keywords:** child-directed marketing, food marketing, food packages, marketing regulation

## Abstract

Food marketing has been identified as a contributing factor in childhood obesity, prompting global health organizations to recommend restrictions on unhealthy food marketing to children. Chile has responded to this recommendation with a restriction on child-directed marketing for products that exceed certain regulation-defined thresholds in sugars, saturated fats, sodium, or calories. Child-directed strategies are allowed for products that do not exceed these thresholds. To evaluate changes in marketing due to this restriction, we examined differences in the use of child-directed strategies on breakfast cereal packages that exceeded the defined thresholds vs. those that did not exceed the thresholds before (*n* = 168) and after (*n* = 153) the restriction was implemented. Photographs of cereal packages were taken from top supermarket chains in Santiago. Photographed cereals were classified as “high-in” if they exceeded any nutrient threshold described in the regulation. We found that the percentage of all cereal packages using child-directed strategies before implementation (36%) was significantly lower after implementation (21%), *p* < 0.05. This overall decrease is due to the decrease we found in the percentage of “high-in” cereals using child-directed strategies after implementation (43% before implementation, 15% after implementation), *p* < 0.05. In contrast, a greater percentage of packages that did not qualify as “high-in” used child-directed strategies after implementation (30%) compared with before implementation (8%), *p* < 0.05. The results suggest that the Chilean food marketing regulation can be effective at reducing the use of child-directed marketing for unhealthy food products.

## 1. Introduction

Childhood obesity is of major worldwide concern, as it is a strong predictor of adult obesity and other health, social, and economic consequences [1]. The development of overweightness and obesity is associated, to a great extent, with the overconsumption of calories [2] and free sugars [3,4]. Unhealthy food and beverage marketing has been identified as a key contributor to childhood obesity [5], as products that are energy dense and high in sugars, such as sugary, sweetened beverages, breakfast cereals, snacks, and candies [6] are often marketed with fun characters, collectible gifts, and other strategies that appeal to children [7,8,9,10]. Because of their limited cognitive and executive skills [7,11,12], children might be especially vulnerable to this type of marketing in advertising [7,8] and on packages [9,10,13,14].

Food packages are of particular concern, as marketing strategies on packaging are an important component of integrated marketing campaigns [7,15] designed to influence consumers at both the point of purchase and during consumption [16,17,18,19,20,21,22]. Packages for sugary and energy-dense products in general [9,22,23], and for sugary breakfast cereals in particular [24,25], have been shown to employ child-directed strategies. These strategies, such as licensed or branded characters, have been shown, in turn, to influence children’s food preferences and choices [19,26], as well as children’s taste perceptions of food [13,27].

Given the significant evidence that child-directed marketing impacts children’s attitudes, preferences, and eating practices [7,8,28,29], the World Health Organization recommended that countries ensure healthier food environments by restricting child-directed marketing of energy-dense and nutrient-poor foods and beverages, particularly products high in saturated fats, sugars, or salt [30]. To date, few countries have implemented statutory child-directed food marketing restrictions, and few studies have assessed those policies [31,32]. Guided by international recommendations to encourage diets that limit a person’s intake of saturated fats, free sugars, and sodium [33], Chile implemented the Food Labeling and Advertising Regulation (Law 20.606) [34,35,36] aimed at preventing childhood obesity through a labeling and marketing restriction on foods above certain defined thresholds in energy, saturated fats, sugars, and sodium [31,37]. The Chilean law, implemented in June 2016, has been considered the most comprehensive regulation of its kind to date [35], due to its wide scope of restrictions on food marketing and its criteria for qualifying foods as “high-in” the above nutrients. Chile also has one of the highest obesity prevalence rates worldwide—24% of children 6–7 years of age [38] and 31% of people ≥15 years of age in Chile are obese [39]. 

The Chilean regulation features progressive cut-off values for energy, saturated fats, sugars, and sodium per 100 g in foods or 100 mL in liquids. Products above these thresholds—“high-in” products henceforth—must use front-of-package warning labels identifying the product as high in the excessive nutrient (products might carry more than one label if they exceed the thresholds for more than one nutrient). “High-in” products also cannot be promoted or sold within schools and are restricted from marketing to children <14 years of age. Products that are not classified as “high-in” are exempted from these restrictions [35,36]. The listed cut-off values only apply to products that contain an ingredient (e.g., added sugar) that increases the content of one or more of the critical nutrients beyond the specified thresholds. Table 1 shows the progression in how these thresholds have been defined, beginning with the least strict thresholds implemented in 2016 to the strictest thresholds implemented in 2019. The present study focuses on the first thresholds implemented in 2016: ≥22.5 g for sugars, ≥6 g for saturated fats, ≥800 mg for sodium, ≥350 kcal in energy density (per 100 g of food). An explanation of the development of these thresholds has been previously published [40]. 

The Chilean law also provides a list of child-directed appeals and incentives that are prohibited from use in the marketing of products that qualify as “high-in” [34,35]. This marketing restriction extends to advertising, as well as product packaging. To our knowledge, this is the first regulation to ban the use of child-directed marketing strategies on product packages. Included in the list of banned strategies is the use of characters, children and child-like figures, cartoons, references to children’s daily lives, gifts, and games. Different from other regulations [41], the Chilean regulation does not exempt trade brands (brand equity characters) from restriction. Therefore, licensed characters and brand characters, such as Tony the Tiger or the Trix Rabbit, are banned from use [13,14,24,42]. However, “high-in” products are allowed to use marketing strategies that are not included in the regulation’s list of child-directed marketing appeals, for example health claims, actors >14 years of age, or emotional appeals suggesting happiness.

The aim of the present study was to assess changes in the prevalence of marketing strategies targeting children in breakfast cereal packages before and after the Chilean regulation, using a repeated cross-sectional design, with one sample of Chilean breakfast cereal packages taken before the 2016 implementation and one sample of cereals taken half a year after the 2016 implementation. Breakfast cereal packages were selected as the focus of this study, given that this product category is documented to be heavily marketed to children [25,43]. This product category was also ideal for assessing the Chilean regulation, because many child-directed cereals have been found to be higher in calories or sugars [24,43,44] compared with their non-child-targeted counterparts. 

The prevalence of child-directed strategies in each period was assessed according to the products’ regulation categorization (“high-in” or “non-high-in”) based on Chile’s first nutrient thresholds. We expected to find a significantly lower proportion of “high-in” breakfast cereal packages with child-directed strategies after the 2016 implementation compared with before this first implementation. We were also interested in examining whether the use of child-directed strategies among packages that did not qualify as “high-in” would change post-implementation, indicating an industry shift from marketing “high-in” cereals to marketing “non-high-in” cereals to children. 

## 2. Materials and Methods

This study examined the use of child-directed marketing strategies in Chilean breakfast cereal packages before and after the June 2016 implementation of the Chilean Food Advertising and Labeling Law. Using a quantitative content analysis of breakfast cereal package photographs, child-directed strategies were identified and categorized by two coders who examined the front, sides, and back of each package. The use of child-directed strategies was analyzed according to regulation categorization (“high-in” or “non-high-in”) and time period (before and after implementation) to assess changes in the prevalence of child-directed strategies before and after the regulation. 

### 2.1. Sample

This study used photographs of breakfast cereal packages taken as part of an ongoing food environment monitoring project conducted by the International Network for Food and Obesity/Non-communicable Diseases Research, Monitoring and Action Support (INFORMAS-CHILE) [45] and the University of Chile’s Nutrition and Food Technology Institute (INTA) [46]. In February–March 2015, before the regulation’s implementation, and again in January–February 2017, post-implementation, six supermarkets in a high-income neighborhood belonging to five of the largest supermarket chains in Santiago (two supermarkets from the same chain) were visited. The selected supermarket chains have stores in all Chilean cities and represent 100% of the market share of supermarkets [47]. An agreement between the Chilean National Association for Supermarkets (ASACH) and the University of Chile’s Nutrition and Food Technology Institute was reached to obtain permission to take pictures in the stores [46]. Supermarkets were selected on the basis of having the greatest variety of products per chain within the neighborhood where photographs were taken. The breakfast cereals photographed in 2015 and 2017 included at least one product from all of the top brands listed in the Euromonitor report of breakfast cereal sales (by retail sales price) per brand in Chile in the years 2015 and 2017 [48].

Trained nutritionists took photographs of all sides of all breakfast cereals encountered, avoiding duplicates [46]. All versions of the same product were collected. In other words, if two products were identical but used different marketing content on their packages, we collected images of both packages. If a product used the same marketing content on packages of different sizes, the photographs of the largest package were taken. Therefore, the nutritionists captured all marketing content for breakfast cereal packages across these supermarkets [46].

A total of 168 Spanish-language breakfast cereals from the 2015 data collection and 146 Spanish-language packages from the 2016 data collection were included in this study. Eight packages at pre-implementation and 25 packages at post-implementation were excluded, because they were not written in Spanish. Across both samples, breakfast cereal photographs included ready-to-eat cereals (*n* = 269), such as flakes, puff, muesli, granola, and fiber cereals, and not-ready-to-eat cereals (*n* = 45), namely traditional and instant oats. 

### 2.2. Coding of Marketing Strategies 

Child-directed food marketing strategies were assessed with a comprehensive protocol that included text and imagery. The protocol was built based on the Chilean food marketing regulation [34], with procedural guidance taken from prior studies of child-directed marketing strategies [7,10,49,50,51,52,53,54]. Packages were coded for two overarching categories of child-directed marketing strategies: the use of child-directed characters and the use of non-character-based elements that appeal to children. These categories are described in greater detail below. Initially, the codebook also included mentions of contests for children and the presence of celebrities. However, only one child-directed contest and one celebrity were found in the sample. Therefore, contests and celebrities were excluded from the analysis. For the remaining elements, the occurrence of at least one marketing appeal within a given strategy (e.g., characters) was coded as the presence of that particular strategy, regardless of the number of times the strategy appeared in the package. 

### 2.3. Child-Directed Characters

Packages were considered to have child-directed characters if they included at least one image of the following on any package face: human youth (i.e., images of people <14 years of age); fantastical non-youth (i.e., human >14 years of age with a superhuman or magical appearance, such as wearing a cape or flying in space); or personified objects (i.e., any anthropomorphized creature or item such as a smiling fruit, spoon, or tiger). For human youth, if it was not clear that a human figure was under 14 years old, coders were instructed to code the figure as a non-youth.

For packages with at least one child-directed character, characters were further categorized as cross-promotional (e.g., at least one character was licensed to entities outside the brand, such as a TV show or movie) or as a sports reference (e.g., at least one character was engaged in sports or physical activity). 

### 2.4. Non-Character Strategies

Packages were considered to have non-character child-directed strategies if they included at least one of the following strategies on any package face: child-oriented gifts (e.g., stickers and toys in or on the package); games (e.g., word searches, puzzles, or other activities on the box); toy references (e.g., depictions of cars, balls, or other items intended for play); school references (e.g., mention of school supplies or images of backpacks or playgrounds); child words (words that specifically reference children, such as “for kids”); and cross-promotions (e.g., names of movies, TV shows, or other brands). Licensed characters were captured under the child-directed characters category.

### 2.5. Coding Reliability

Two Chilean coders trained for two weeks on how to apply the codebook to products outside the samples used in this study. Then, both coders analyzed a random selection of 23% of the sample (*n* = 73) to assess inter-rater reliability. Simple percentage of agreement and two-rater chance-corrected agreement coefficient Gwet’s AC2 were calculated with AgreeStat 2015.6.1 [55]. Percentage of agreement for individual codes ranged from 92.6% to 100% and Gwet AC2 ranged from 0.92 to 1. The code with the lowest (92.6%) agreement pertained to the presence of “non-youth doing sports” and was intended to be used in connection with the “fantastical non-youth” code to identify images of adults or teens who were depicted as superhuman or magical through exaggerated sports or exercise performances. However, the combination of “fantastical non-youth” and “non-youth doing sports” never occurred within the sample, and so, this disagreement made no impact on the analysis. The remaining codes had a percentage of agreement of at least 98%. Among those few instances of disagreement, we randomly selected which coder’s decision would be included in the final dataset.

### 2.6. Product Categorization

Trained nutritionists recorded total sugars (g)/100 g, saturated fats (g)/100 g, sodium (mg)/100 g, and energy (kcal)/100 g for each product using package nutrition facts panel data collected as part of the INFORMAS-CHILE project [45]. Eight packages did not provide information for total sugars, in which case, total sugars were imputed from similar products collected during the same year with a similar list (and order) of ingredients. A different brand was used only if the same brand was not available. For not-ready-to-eat cereals, nutrient content and energy were calculated based on 100 g of reconstituted product. Packages in the pre-implementation and post-implementation samples were categorized as “high-in” (=1) if they exceeded any of the 2016 nutrient thresholds in sugars, sodium, saturated fats, or energy, provided they contained an ingredient that increased the natural content of the given critical nutrient [35]. For the pre-implementation sample, a categorization of “high-in” was given to products that exceeded at least one of the June 2016 nutrient thresholds and would be subject to regulation if the thresholds were in effect at that time. For the post-implementation sample, a “high-in” categorization was given to packages that were regulated at that time for exceeding at least one of the 2016 thresholds. Any packages that did not exceed a threshold in the pre- or post-implementation sample were categorized as “non-high-in” (=0).

### 2.7. Analysis

Crosstabulations were used to examine the proportion of “high-in” vs. “non-high-in” breakfast cereal packages at pre- vs. post-implementation that used child-directed characters, non-character strategies, and the specific marketing elements described within the character and non-character strategy categories. Fisher’s exact tests were used to evaluate whether differences in proportions found in the crosstabulations were statistically significant. A logistic regression was used to test whether regulation status (“high-in” vs. “non-high-in”) interacted with time period (pre- vs. post-implementation) to predict the presence of child-directed strategies in breakfast cereal packages. Post-hoc logistic regressions were performed to interpret the significant interaction found between regulation status and timeframe. All analyses were performed using STATA/SE 16.0.

## 3. Results

Table 2 describes the number and percentage of products categorized as “high-in” or “non-high-in” at pre- and post-implementation, showing the quantity of “high-in” products that exceeded sugars, saturated fats, sodium, and energy thresholds. About 79% of products at pre-implementation were categorized as “high-in”, 98% of which exceeded levels for calories, 66% for sugars, and 64% for both calories and sugars. At post-implementation, 59% of the products were categorized as “high-in”, 98% of which were high in calories, 59% high in sugars, and 58% high in both calories and sugars.

Table 3 shows the percentage of packages using child-directed strategies before and after implementation, according to regulation categorization. Not shown in Table 3, all child-directed strategies were found in ready-to-eat breakfast cereals at pre- and post-implementation. As shown in Table 3, the percentage of packages overall that used at least one child-directed strategy was significantly lower after implementation (21%) compared with before implementation (36%), *p* < 0.05. This difference was primarily due to the lower number of packages using non-character strategies in the post-implementation sample, *p* < 0.05. 

Although the total percentage of packages that specifically used a character decreased from 30% to 21% after implementation, this difference was not statistically significant, *p* = 0.07. In fact, the use of characters, personified objects in particular, was the most prevalent child-directed strategy used in breakfast cereal packages at pre- and post-implementation. However, among those packages with characters, the total percentage of packages using a character that was licensed significantly decreased from 13% to 0.8% after implementation, as did the percentage of packages featuring physically active characters (from 5% to 0%), *p* < 0.05. 

Among packages categorized as “high-in”, the prevalence of packages with at least one child-directed strategy significantly decreased from 43% at pre-implementation to 15% at post-implementation, *p* < 0.05. The use of characters decreased significantly from 36% of “high-in” packages at pre-implementation to 15% of “high-in” packages at post-implementation, *p* < 0.05. The use of non-character strategies also decreased significantly from 23% at pre-implementation to 0% at post-implementation, *p* < 0.05.

Among packages categorized as “non-high-in”, the prevalence of packages using at least one child-directed strategy was significantly higher, rising from 8% before implementation to 30% after implementation, *p* < 0.05. Both the use of characters (from 8% to 28%) and the use of non-character strategies (from 0% to 10%) increased significantly within “non-high-in” packages, *p* < 0.05. It is worth noting that no packages categorized as “non-high-in” in the pre-implementation sample used personified objects, yet the use of personified objects became the most prevalent child-directed strategy within the post-implementation sample of “non-high-in” packages.

From the initial logistic regression, the pre-/post-implementation timeframe significantly interacted with the product regulation status (“high-in” vs. “non-high-in”) to predict whether breakfast cereal packages were likely to feature child-directed strategies, χ^2^(3) = 30.51, *p* < 0.001, Odds Ratio (OR): 0.05, 95% CI: 0.011, 0.217, *p* < 0.001. Table 4 shows the results of the post-hoc analyses used to further interpret this interaction. At pre-implementation, “high-in” products were significantly more likely to feature child-directed strategies compared with “non-high-in” products, OR: 8.36, 95% CI 2.44, 28.63. At post-implementation, “high-in” products were significantly less likely to use child-directed strategies compared with “non-high-in” products, OR: 0.416, 95% CI 0.19, 0.93. These post-hoc findings correspond with the observations (see Table 3) that 43% of “high-in” packages vs. 8% of “non-high-in” packages contained a child-directed strategy at pre-implementation, whereas 15% of “high-in” packages and 30% of “non-high-in” packages featured a child-directed strategy at post-implementation.

## 4. Discussion

This study compared the prevalence of child-directed strategies in breakfast cereal packages in a sample of products collected before the Chilean food marketing regulation was implemented with a similar sample collected after implementation. The Chilean regulation is the only statutory regulation to date that restricts child-directed food marketing on unhealthy food packages [31] and provides the most comprehensive list of strategies and techniques defining child-directed marketing for policy implementation and evaluation purposes [35]. 

We found that the prevalence of breakfast cereal packages using child-directed strategies decreased overall after the regulation’s implementation, driven by the significant reduction in the prevalence of child-directed strategies used by breakfast cereals qualifying as “high-in” according to the first implemented nutrient thresholds. Before implementation, cereals categorized as “high-in” for exceeding regulation-defined thresholds in sugars, saturated fats, sodium, and/or calories per 100 g were more likely to have child-directed strategies (43%) compared with cereals categorized as “non-high-in” (8%). After the regulation’s first implementation, this relation was reversed. At post-implementation, a larger percentage of “non-high-in” products used child-directed strategies (30%) compared with “high-in” products (15%). When combining “non-high-in” and “high-in” products, the total percentage of packages using child-directed strategies dropped from 36% before the June 2016 implementation to 21% after implementation.

Although we expected to find a decrease in child-directed marketing in “high-in” cereals and anticipated a potential increase in child-directed marketing in “non-high-in” cereals, we are unable to discern from our data what specific causes explain this shift. We did observe that the majority of “non-high-in” packages with child-directed strategies that we found in the post-implementation sample previously had higher sugar levels and had since been reformulated to fall just below the threshold. In those cases, the products changed their nutritional composition rather than their marketing strategies. More research is needed to understand the extent of reformulation as a possible strategy for retaining the use of child-directed strategies across product categories. 

As packaging is a predominant medium through which children are exposed to food marketing [23], the reduction in child-directed strategies found in this study suggests that children in Chile are being less exposed to child-directed marketing of products high in calories or sugar in their food environment. This is highly relevant because child-directed marketing of unhealthy food has been associated with obesity development [8]. We therefore interpret our findings as evidence that Chile’s Food Labeling and Advertising Regulation is a promising tool for reducing children’s exposure to child-directed marketing on unhealthy packaged foods. 

Our findings indicate a slightly lower prevalence of the use of child-directed strategies before the regulation’s implementation compared with research from other countries that focus on ready-to-eat cereals [24,44]. In our pre-implementation sample, we found that more than one-third (36%) of breakfast cereal packages, including ready-to-eat and not-ready-to-eat cereals, used at least one child-directed strategy. This percentage increases to 43% if only ready-to-eat cereals are considered. This percentage is lower than the 46% and 51% of packages using child-directed strategies found for ready-to-eat breakfast cereals in studies conducted in the United States (US) [44] and Guatemala [24], respectively. It is possible that our sample captured a wider range of products and therefore might have captured more products that do not use child-directed strategies compared with the cited studies. The US study collected data only from the four main breakfast cereal manufacturers in the US, and the Guatemalan study collected all ready-to-eat breakfast cereals available in the one supermarket representing its largest supermarket chain located in a middle-to-high socioeconomic status urban sector. Our sample included all different breakfast cereals available in six different supermarkets from five different chains in a high-income neighborhood of urban Santiago. It is possible that our sample might include more products that did not feature child-directed marketing due to a greater variety of products captured, differences in how products might have been marketed in high-income urban supermarkets vs. other venues, or differences in definitions of what constituted child-directed marketing. Note that our definitions of child-directed marketing were specific to evaluating the Chilean regulation, and therefore, we excluded other appeals that might be attractive to children but that were not listed as appeals subject to restriction.

If we only focus on the prevalence of products featuring child-directed characters at baseline, the prevalence we found (29%) is higher than the prevalence (21%) reported in a study in New Zealand [56]. In their study, all breakfast cereals available for purchase (*n* = 247), including ready-to-eat and non-ready-to-eat cereals, were recorded in two major supermarkets in Auckland in 2013. This sample collected in Auckland is markedly larger than our baseline sample. Therefore, it is possible that the New Zealand study captured an even greater variety of products than we did, and to the extent that they captured a greater variety of products that were not aimed toward children in their larger sample, it is not surprising that the percentage of characters they found would be lower than the percentage we found. It is also possible, of course, that the prevalence of child-directed strategies varies from country to country, according to local market characteristics.

Consistent with reports across other countries [6], our study found the use of characters to be the dominant child-directed strategy on the child-directed packages we analyzed. Even after Chile’s regulation was first implemented, we continued to find child-directed characters on “high-in” breakfast cereal packages, albeit the prevalence of these characters was significantly lower among “high-in” packages compared with the baseline. In fact, the use of characters was the only child-directed strategy that persisted among “high-in” packages after implementation. At the same time, we found that “non-high-in” products were using more characters at post- than at pre-implementation. As a result, characters remained the most used child-directed strategy across all breakfast cereals. This finding highlights the key role that characters play in breakfast cereal’s child-directed marketing and supports the development of regulations that limit the extent to which “high-in” products use these highly child-attractive strategies [13,42,57,58]. 

To date, 16 countries have implemented statutory regulations to restrict unhealthy food marketing to children. The most common regulations are partial restrictions on unhealthy food advertising on television and restricted food promotions in schools. Unfortunately, the few policies that have been assessed have shown little to no reduction in unhealthy food advertising [31]. Package marketing restrictions have only been implemented in Chile, despite evidence showing that point-of-sale promotions and marketing on packages have a significant influence on children’s food preferences and choices [13,18,19,21]. According to our findings, after the food marketing regulation implementation, we found that 85% of packages were compliant with the child-directed marketing restriction and 15% (*n* = 13) of “high-in” packages did not comply with the restriction due to their continued use of child-directed characters. This percentage of noncompliance is similar to the 18% noncompliance across food marketing (including advertising and packaging) reported by the Chilean Ministry of Health (MINSAL) during the first year of the regulation’s implementation [59]. 

Our findings support the adaptation and implementation of this type of intervention in other countries interested in developing regulations to protect children from the influence of unhealthy food marketing. However, our findings also highlight the challenges in obtaining 100% compliance. One major consideration that might explain noncompliance is the application of Chile’s restriction to brand characters. To our knowledge, this is the first regulation that bans the use of child-directed characters without exemptions. Other countries with regulations that ban the use of child-directed characters, such as the United Kingdom (UK), exempt brand equity characters from their restriction [41]. During the initial period of inspection in Chile, companies challenged noncompliance sanctions in court, citing concerns about brand equity and a need to defend the intellectual property rights of their trademarks as reasons for needing a judicial remedy for the sanction [59]. However, MINSAL argued that the law prohibits any food advertising aimed at children under 14 years of age, irrespective of whether these child-directed promotion strategies were registered trademarks [59]. 

We must also note that the product photographs collected for our post-implementation sample were taken in the sixth and seventh months after the regulation was implemented. During this timeframe, MINSAL was mainly giving reprimands and a deadline to comply with the new regulation, although on a few occasions, MINSAL prohibited the sale of entire product lines that featured child-directed characters. As such, the noncompliant products reported in this study may be understood as part of the process of adaptation to the new regulated context. Thus, our study is limited to the timeframes in which package photographs were taken from the six sampled supermarkets and the variety of marketing strategies present on the breakfast cereal packages available in those months within 2015 and 2017. We cannot claim that the photographs analyzed constitute a nationally representative sample of breakfast cereals packages. However, the group of photographs we analyzed did capture at least one product of all the breakfast cereal brands listed in the Euromonitor report of sales per brand in Chile in the years 2015 and 2017 [48]. Future studies would be needed to examine all breakfast cereals available in the Chilean market to fully document the prevalence of child-directed strategies in this product category. 

In addition, other product categories should also be examined to evaluate the impact of the regulation for other types of foods and beverages. Sugar-sweetened beverages might be of particular interest, given that the per capita sugar-sweetened beverage sales in Chile are the highest worldwide [60,61]. One study of beverage packaging in Chile before the regulation was first implemented noted that beverages featuring child-directed characters had higher sugar and energy levels than beverages that did not feature those child-directed strategies on their packages [10]. Future work could examine changes in marketing strategies, as well as reformulation, among beverages sold in Chile after the regulation. 

As noted above, our study is also limited in its focus on child-directed strategies banned by the Chilean regulation [34]. Thus, our findings support the conclusion that regulations with defined lists of child-directed content strategies can be effective at reducing those defined child-directed marketing strategies on packages. However, we acknowledge there are other known marketing strategies that can be appealing to children. For example, the regulation does not include restrictions on general fun appeals, images of adolescents or teens, and design techniques, such as unconventional colors and fonts, which are all strategies that are commonly used to target children [14,15,19,62]. For example, research shows that emotional strategies, such as fun or happiness, are effective across age groups at influencing attitudes and behaviors toward brands and products by creating an association between the positive emotion and the brand [63]. Images of youth >14 years of age might be highly influential models to older children, especially given their susceptibility to peer norms and aspirational images [64,65]. The Chilean regulation only restricts strategies that were deemed by MINSAL to clearly reference or target young children, allowing the use of strategies that might have a wider appeal across age ranges. This is a limitation of the regulation itself. To fully evaluate all possible appeals that might attract children to unhealthy foods and beverages, it is important to find ways to monitor marketing strategies that are not covered by this regulation. Using a more comprehensive concept, such as “child-appealing”, rather than “child-directed”, marketing would broaden the scope of this type of study and assist in examining the limits of this regulation.

Additionally, the Chilean regulation does not ban the use of health- and nutrition-related claims in “high-in” products, with the exception that products cannot contradict the warning label they carry, e.g., claiming low sugar when they qualify for a “high in sugar” label [34,35]. Even though health claims are not specifically child-directed, there is evidence of a health halo effect associated with the use of health claims in food advertising, leading young [66,67] and adult consumers [68,69,70,71] to perceive the entire product as being healthy, regardless of its overall nutritional quality. This health halo effect is problematic, given evidence that health claims are widely used in products high in sugars, sodium, fats, or calories [9,72,73]. Further research is needed to evaluate the changes in the prevalence of these strategies and the potential these strategies have on children’s attitudes and preferences toward food products that carry warning labels [66,67].

Finally, given that the regulation has three different nutrient thresholds, which progressively become stricter over time, further research is needed to assess compliance at these different stages of implementation. Likewise, a broader analysis of the changes in marketing strategies used to promote regulated and unregulated food products is needed across these different stages. It remains to be seen how the reduction of child-directed strategies in food packages are reflected in the population’s food attitudes and preferences or if these changes generate the intended improvement on Chilean children’s diets and health. Based on the extant literature [1,8,74], the changes in breakfast cereal marketing we have shown in this study have promising potential for contributing to an eventual positive impact on attitudinal, behavioral, and health indicators. 

## 5. Conclusions

The present study assessed the changes on breakfast cereal packages’ marketing strategies after the implementation of the first statutory regulation of child-directed marketing, which limits marketing content on food packages. Our study found that 85% of “high-in” breakfast cereal packages were compliant with the child-directed marketing restriction seven months after the regulation was implemented, showing a significant reduction of child-directed marketing in products with high levels of critical nutrients and calories. In contrast, after the regulation was implemented, “non-high-in” products with child-directed strategies were significantly more prevalent than before the implementation. These findings reflect the scope of the Chilean regulation with respect to its ability to change the prevalence of child-directed marketing strategies on food packages. 

## Figures and Tables

**Table 1:** Progressive thresholds for defining “high-in” foods in the Chilean law of food labeling and advertising.

Critical Nutrient	Nutrient Threshold per 100 g of Food		
	June 2016	June 2018	June 2019
Energy (kcal)	≥3.5	≥3	≥2.75
Sodium (mg)	≥800	≥500	≥400
Total sugars (g)	≥22.5	≥15	≥10
Saturated fats (g)	≥6	≥5	≥4

**Table 2:** Descriptive statistics for “high-in” and “non-high-in” breakfast cereal packages sampled pre- and post-implementation.

Critical Nutrient	Pre-Implementation (*n* = 168)					Post Implementation (*n* = 146)				
	“non-high-in” *n* = 36 (21.4%)		“high-in” *n* = 132 (78.6%)			“non-high-in” *n* = 60 (41%)		“high-in” *n* = 86 (59%)		
	Content per 100 g of food		Content per 100 g of food		*n* (%) of “high-in” packages above specific nutrient threshold	Content per 100 g of food		Content per 100 g of food		*n* (%) of “high-in” packages above specific nutrient threshold
	*Mdn*	*Min–Max*	*Mdn*	*Min–Max*		*Mdn*	*Min–Max*	*Mdn*	*Min–Max*	
Energy (kcal)	326	46.5–395	380	101.3–465	130 (98)	339	41.2–400	384	89.7–465	85 (98)
Sugars (g)	1.78	0–22.5	26	0-40	87 (66)	9.7	0–22.2	27.2	0–40	51 (59)
Saturated fats (g)	0.75	0–3.9	1.4	0–7.4	2 (2)	1.1	0.16–4.6	1.35	0–7.4	1 (1)
Sodium (mg)	78.2	0.3–604	184	4.9–689	0	66.6	0.7–430	150.5	2.8–585	0

“High-in” products are products exceeding at least one 2016 regulation-defined threshold in sugars, saturated fats, sodium, or energy. Products “high-in” for both calories and sugars *n* = 85 (64% of “high-in” products) at pre-implementation and *n* = 50 (58% of “high-in” products) at post-implementation. *Mdn:* Median. *Min–Max:* Minimum and maximum values. *n* (%): Number (percentage).

**Table 3:** Differences in the percentage of packages using child-directed strategies within “high-in” and “non-high-in” breakfast cereals at pre- versus post-implementation.

Type of Child-Directed Strategy on Package	Percentage of “Non-High-In” Packages with at Least One Child-Directed Strategy			Percentage of “High-In” Packages with at Least One Child-Directed Strategy			Percentage of Total Packages Sampled with at Least One Child-Directed Strategy		
	Pre-(2015)	Post-(2017)	Difference (2017–2015)	Pre-(2015)	Post-(2017)	Difference (2017–2015)	Pre-(2015)	Post-(2017)	Difference (2017–2015)
	(*n* = 36)	(*n* = 60)		(*n* = 132)	(*n* = 86)		(*n* = 168)	(*n* = 146)	
Any child-directed strategy	8.33	30.00	21.67 *	43.18	15.12	−28.07 *	35.71	21.23	−14.48 *
Any character strategy	8.33	28.33	20.00 *	35.61	15.12	−20.49 *	29.76	20.55	−9.21
Personified object	0.00	21.67	21.67 *	30.30	13.95	−16.35 *	23.81	17.12	−6.69
Human youth	8.33	6.67	−1.67	6.82	1.16	−5.66	7.14	3.42	−3.72
Fantastical non-youth	0.00	0.00	0.00	3.03	0.00	−3.03	2.38	0.00	−2.38
Licensed?	0	0.76	0.76 *	13.33	0	13.33 *	13.33	0.76	−12.57 *
Doing exercise?	0	0	0	5.3	0	5.3 *	5.3	0	−5.3 *
Any non-character strategy	0.00	10.00	10.00 *	23.48	0.00	−23.48 *	18.45	4.11	−14.34 *
School references	0.00	0.00	0.00	3.79	0.00	−3.79	2.98	0.00	−2.98
Toy references	0.00	1.67	1.67 *	7.58	0.00	−7.58 *	5.95	0.68	−5.27 *
Children words	0.00	5.00	5.00 *	8.33	0.00	−8.33 *	6.55	2.05	−4.49
Child-oriented gifts	0.00	3.33	3.33	1.52	0.00	−1.52	1.19	1.37	0.18
Games	0.00	6.67	6.67 *	9.09	0.00	−9.09 *	7.14	2.74	−4.40
Cross-promotions	0.00	3.33	3.33	4.55	0.00	−4.55	3.57	1.37	−2.20

Regulated products are products exceeding at least one 2016 regulation-defined threshold in sugars, saturated fats, sodium, or energy. Comparisons of proportions of breakfast cereal packages featuring the given child-directed strategy in the 2015 versus 2017 samples made using Fisher’s exact test. * *p* < 0.05. All coded types of child-directed strategies were present on at least one ready-to-eat breakfast cereal.

**Table 4:** Likelihood of breakfast cereal packages featuring child-directed marketing strategies pre- and post-implementation.

Pre- vs. Post-Implementation	Prevalence of Products Using at Least One Child-Directed Strategy		Logistic Regression OR (95% CI)	*p*-Value
	“non-high-in”	“high-in”		
Pre-implementation	8.33% (3/36)	43.18% (57/132)	8.36 (2.44, 28.63)	<0.01
Post-implementation	30% (18/60)	15.12% (13/86)	0.416 (0.19, 0.93)	<0.05

Findings from the interpretation of significant interaction of timeframe X regulation categorization predicting the presence of at least one child-directed strategy on breakfast cereal packages, χ²(3) = 30.51, *p* < 0.001, OR: 0.05, 95% CI: 0.011, 0.217. “high-in” products are products exceeding at least one 2016 regulation-defined threshold in sugars, saturated fats, sodium, or energy. CI: Confidence Interval OR: Odds ratio. Note: Bold font indicates statistical significance.
